# Image-Based Recognition of Children’s Handwritten Arabic Characters Using a Confidence-Weighted Stacking Ensemble

**DOI:** 10.3390/s25247671

**Published:** 2025-12-18

**Authors:** Helala AlShehri

**Affiliations:** Computer and Information Technology Department, Jubail Industrial College, Jubail 35718, Saudi Arabia; shehrihel@rcjy.edu.sa

**Keywords:** deep learning, machine learning, Arabic character recognition, child handwriting, ensemble learning, confidence thresholding, convolutional neural networks

## Abstract

Recognizing handwritten Arabic characters written by children via scanned or camera-captured images is a challenging task due to variations in writing style, stroke irregularity, and diacritical marks. Although deep learning has advanced this field, building reliable systems remains challenging. This study introduces a stacking ensemble framework for sensor-acquired handwriting data, enhanced with a dynamic confidence-thresholding mechanism designed to improve prediction reliability. The framework integrates three high-performing convolutional neural networks (ConvNeXtBase, DenseNet201, and VGG16) through a fully connected meta-learner. A key feature is the use of an optimized threshold that filters out uncertain predictions by maximizing the macro $F_{1}$ score on validation data. The framework is evaluated on two benchmark datasets for children’s Arabic handwriting: Hijja and Dhad. The results demonstrate state-of-the-art performance, with an accuracy of 95.13% and $F_{1}$ score of 94.62% on Hijja and an accuracy of 96.14% and $F_{1}$ score of 95.59% on Dhad. Compared to existing methods, the proposed approach achieves more than a 3% improvement in Hijja accuracy while maintaining robust performance across diverse character classes. These findings highlight the effectiveness of confidence-based stacking ensembles in enhancing reliability for Arabic handwriting recognition and suggest strong potential for automated educational assessment tools and intelligent tutoring systems.

## 1. Introduction

Automatic handwriting recognition (AHR) plays an important role in education, digital archiving, and intelligent document processing [1]. Sensor-captured handwriting from scanners, cameras, or touch devices often contains noise and distortion, which make recognition challenging. AHR systems convert handwritten input, whether offline or online, into digital text for automated use [2,3,4].

Arabic handwriting adds further complexity. The script contains 28 characters written in a semi-cursive, right-to-left form, and each character changes shape depending on its position within a word [5]. Table 1 shows how a letter such as Ayn (ع) appears in several positional forms. Many Arabic characters also share similar base shapes and differ only in diacritical marks, which create high inter-class similarity [6]. The examples include ث/ت/ب and خ/ح/ج. These properties render Arabic handwriting recognition, especially from sensor-derived images, more difficult than Latin scripts [7].

Children’s handwriting introduces additional challenges due to immature motor control and limited writing experience. This often results in irregular strokes, non-standard proportions, and incomplete character shapes. Although interest in educational technologies is growing, few studies focus on Arabic handwriting produced by children. The Hijja and Dhad datasets support research in this area, yet baseline CNN performance remains modest because of the high intra-class variability in children’s writing.

The modest performance of baseline CNNs on these datasets, driven by high intra-class variability, motivates the need for more robust modeling approaches. A stacking ensemble integrates complementary strengths from multiple CNN architectures and produces more stable predictions than simple averaging or majority voting. Transformer models typically require larger datasets and higher-resolution inputs, which limit their effectiveness for isolated Arabic characters at 32 × 32 resolution. At this scale, most discriminative information exists at the local stroke level and is captured more effectively by CNNs.

This study proposes a deep learning system for recognizing children’s Arabic handwritten characters using a stacking ensemble of multiple CNNs. The framework integrates three strong CNN models with softmax outputs that are combined through a fully connected meta-learner. A dynamic confidence-thresholding mechanism improves reliability by selecting the threshold that maximizes the macro-F1 score and filtering uncertain predictions.

Softmax-level fusion is selected because the base CNNs produce heterogeneous and non-aligned latent features, which render feature-level concatenation prone to overfitting. Logit-level fusion introduces calibration inconsistencies, whereas softmax outputs provide normalized probability distributions that the meta-classifier can learn effectively. The use of 32 × 32 resolution aligns with prior benchmarks and preserves critical diacritical and stroke information while avoiding unnecessary background noise and computational cost.

Unlike prior studies that rely on a single CNN or handcrafted features, the proposed framework integrates diverse learned representations and incorporates prediction reliability into the decision process. This combination results in a more robust and dependable solution for children’s Arabic handwriting recognition.

In summary, the contributions of this study are as follows:

1. A Confidence-Based Stacking Ensemble Framework: We introduce an ensemble that strategically integrates three high-performing CNN architectures (ConvNeXtBase, DenseNet201, and VGG16) through a fully connected meta-learner.
2. A Dynamic Confidence-Thresholding Mechanism for Reliable Predictions: We propose a threshold optimization technique that maximizes the macro-F1 score on validation data. This mechanism filters out low-confidence predictions, thereby significantly improving the reliability and practical deployability of the system for processing real-world, noisy sensor-derived images.
3. A Comprehensive Benchmark of Modern CNNs for Arabic Script: We conduct an extensive empirical evaluation of multiple state-of-the-art architectures (including ConvNeXt, DenseNet, EfficientNet, VGG, and ResNet) on children’s handwritten Arabic characters, providing a valuable reference for model selection in this domain based on both accuracy and F1 score.
4. Rigorous Validation Demonstrating Superior Robustness: The proposed framework is rigorously evaluated on two challenging, publicly available datasets (Hijja and Dhad), achieving state-of-the-art results. It demonstrates consistent and robust performance across varied writing styles and noise levels commonly encountered in sensor-captured data.
5. In-depth error analysis providing actionable insights: Beyond aggregate metrics, we provide a detailed per-class performance analysis. This reveals recurring challenges in distinguishing visually similar letter pairs (e.g., غ/ع, ذ/د), offering clear guidance for future research focused on resolving fine-grained visual ambiguities.

## 2. Related Research

Arabic handwritten character recognition has been extensively explored in recent years, driven by advances in deep learning and the growing availability of benchmark datasets. Early studies focused on general recognition tasks using adult handwriting, leveraging convolutional neural networks (CNNs) to extract spatial features from complex Arabic scripts. Several studies have reported strong results on standard datasets. For instance, Sousa proposed a hybrid training algorithm combining adaptive and stochastic gradient descent for recognizing Arabic characters and digits using an ensemble of CNNs. The approach achieved state-of-the-art results on the MADbase (99.74% validation; 99.47% test) and AHCD (98.60% validation; 98.42% test) datasets [8]. Similarly, Boufenar et al. applied transfer learning with deep CNNs to the OIHACDB and AHCD datasets, reporting superior accuracy compared to traditional approaches [9]. Alyahya et al. introduced an ensemble framework built upon an adapted ResNet-18 model with dropout layers, which attained a top accuracy of 98.30% on AHCD [10].

Hybrid approaches incorporating neural networks and other machine learning models have also demonstrated significant potential. Ali and Mallaiah developed a CNN and support vector machine (SVM) model incorporating dropout regularization and a max-margin minimum classification error loss function, yielding strong performance across datasets such as AHDB, AHCD, HACDB, and IFN/ENIT [1]. Ullah and Jamjoom highlighted the role of batch normalization and dropout in improving generalization, achieving 96.78% accuracy on AHCD [11]. Further, Alrobah, and Albahli combined CNN-extracted features with SVM and XGBoost classifiers, reporting 96.3% accuracy on the Hijja dataset [12].

To address challenges such as data imbalance, Nayef et al. proposed an optimized leaky ReLU activation combined with batch normalization, achieving up to 99% accuracy on AHCD and MNIST and 90% on Hijja [13]. Wagaa et al. examined several optimization algorithms and employed data augmentation with dropout regularization, reaching 98.48% and 91.24% accuracy on AHCD and Hijja, respectively [14]. Bouchriha et al. addressed character shape variations through a specialized CNN model, which achieved 95% accuracy on the Hijja dataset, underscoring the efficacy of structure-aware models [15].

In contrast, limited research has addressed the recognition of Arabic characters written by children, despite its growing relevance in educational applications. To mitigate the shortage of child-focused handwriting datasets, Altwaijry and Alturaiki introduced Hijja, a dataset comprising 47,434 Arabic characters written by 591 children aged 7–12 [16]. They proposed a CNN-based recognition model trained on both Hijja and AHCD datasets, achieving 97% accuracy on AHCD and 88% on Hijja, thereby highlighting the challenges of recognizing children’s Arabic handwriting. Alwagdani and Jaha developed a CNN model augmented with handcrafted features to distinguish between adult and child handwriting, achieving 93% accuracy on children’s characters and 94% in writer-group classification using the Hijja and AHCD datasets [17]. Alheraki et al. introduced a custom CNN approach that incorporated a multi-model strategy based on stroke count and dataset fusion, achieving 91% on Hijja and 97% on AHCD, with an average performance of 96% [18].

Durayhim et al. compared a custom CNN with a pre-trained VGG-16 model, showing that their custom architecture yielded superior results on both the Hijja and AHCD datasets [19]. AlMuhaideb et al. introduced the Dhad dataset, which was created specifically for children aged 7–12, and benchmarked several deep learning models, including MobileNet, ResNet50, and DenseNet121 [20].

In summary, existing studies on Arabic handwritten character recognition have achieved strong performance using individual CNN architectures, handcrafted features, or hybrid learning pipelines. However, most prior research has focused on adult handwriting, and very few studies have addressed the unique variability and irregularity found in children’s writing. Additionally, earlier approaches rarely incorporated ensemble strategies or uncertainty-aware mechanisms to enhance reliability. These gaps highlight the need for a robust, confidence-aware framework capable of leveraging complementary model strengths while handling ambiguous or low-quality samples, which motivates the method proposed in this study.

## 3. Methodology

This section describes the techniques and methods used to build the proposed system for detecting child handwritten characters.

### 3.1. Datasets

In this study, we used two publicly available datasets to evaluate the performance of our system: the Hijja dataset [16] and the Dhad dataset [20].

The Hijja dataset consists of 47,434 handwritten Arabic characters collected from 591 children aged 7 to 12 years. Each participant provided samples of all 28 Arabic letters, as well as one additional class for the Hamza character. The dataset was divided into training and testing subsets using an 80–20 partition, with 37,933 samples for training and 9501 samples for testing.

The Dhad dataset is a recently introduced resource focusing on children’s handwritten Arabic characters. It contains a total of 55,587 samples encompassing all 28 Arabic letter classes in their various forms with the *Hamza* character. These samples were collected from multiple children between the ages of 7 and 12 years old. The dataset was partitioned into 60% for training, 20% for validation, and 20% for testing.

**Figure 1:**
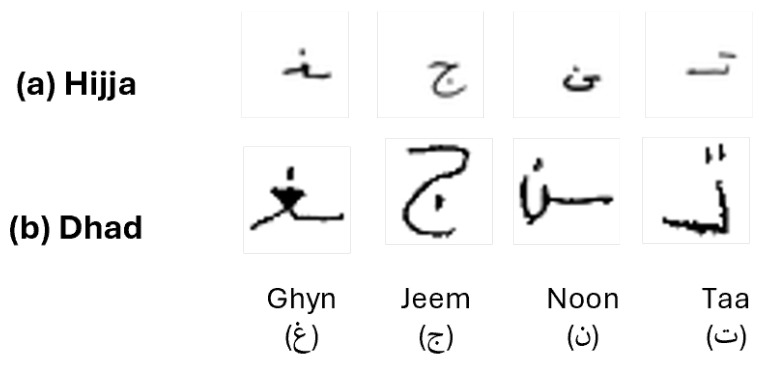
Examples of children’s handwritten Arabic characters from the Hijja (**a**) and Dhad (**b**) datasets, rendered in a 32×32 pixel grid. The characters typically occupy a bounding region of approximately 12–16 pixels in width and 12–18 pixels in height, with stroke widths of about 1–2 pixels. This provides contextual information about the scale of handwriting relative to the fixed input resolution.

Both datasets present unique challenges due to the diversity of writing styles and variations in sample quality, necessitating the development of robust algorithms for effective recognition. The letters were written in both isolated and connected forms, depending on their positions at the beginning, middle, or end of a word. The characters are provided as grayscale images in both datasets, each standardized to a size of 32 × 32 pixels. Figure 1 shows samples of children’s handwritten Arabic characters from both the Hijja and Dhad datasets. The choice of these datasets allows for a comprehensive evaluation of the proposed system’s ability to handle the complexities of Arabic handwriting, with a specific focus on the challenges posed by children’s writing styles.

### 3.2. Proposed Method

**Figure 2:**
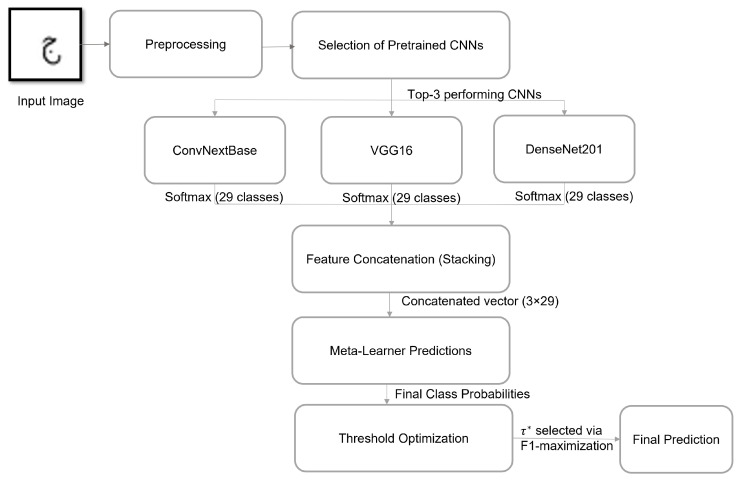
Overview of the proposed Arabic handwritten character recognition framework using stacking ensemble and confidence thresholding.

The proposed system follows a thorough multi-stage pipeline that includes data preprocessing, selection of base CNN models, ensemble learning through stacking, and dynamic threshold optimization, as shown in Figure 2. The process begins with image processing and data augmentation techniques. A thorough evaluation of multiple state-of-the-art convolutional neural network (CNN) architectures was conducted to identify the models most suited for the recognition task. The top three performing models were selected as base learners in a stacking ensemble framework. Each model independently generates softmax probability outputs, which are concatenated and passed to a meta-learner, a fully connected neural network trained to learn the optimal combination of predictions. To further improve prediction reliability, a confidence threshold τ was applied to filter low-confidence outputs, with the optimal value $\tau^{*}$ determined automatically by maximizing the macro-F1 score on the validation set. The subsequent subsections provide a detailed description of each component in this process.

#### 3.2.1. Preprocessing

Prior to model training, a unified preprocessing pipeline was applied to ensure consistency across both datasets and prepare the images for subsequent model processing. All samples were standardized to a 32 × 32 × 3 RGB format. Dhad images (PNG) were loaded in grayscale, converted to RGB, resized to 32 × 32 using bilinear interpolation, and normalized. Hijja samples (CSV) were reshaped to 32 × 32, inverted to match the white-background style of Dhad, and expanded to RGB through channel duplication. The 32 × 32 resolution was retained because it is the native resolution of the Hijja dataset and widely adopted in prior Arabic handwriting studies, ensuring methodological comparability.

To enhance generalization and simulate natural variation in children’s handwriting, a data augmentation pipeline was applied, including random zooming, rotations within $\pm 20^{\circ }$, and controlled horizontal and vertical flipping. These transformations introduced variability in scale, orientation, and writing direction, helping the model learn robust representations despite inconsistencies in handwritten character formation.

#### 3.2.2. Transfer Learning with Pre-Trained CNN Models

To effectively recognize handwritten Arabic characters while minimizing training complexity, the proposed system leverages transfer learning through a diverse set of pre-trained convolutional neural network (CNN) architectures. Transfer learning allows the reuse of feature representations learned from large-scale datasets such as ImageNet, which are then fine-tuned for the target task. This strategy reduces the reliance on extensive labeled data, accelerates training, and improves model performance.

A variety of state-of-the-art convolutional neural network (CNN) architectures were evaluated for their effectiveness in recognizing handwritten Arabic characters, including ConvNeXtBase, DenseNet201, VGG16, EfficientNet, MobileNet, ResNet50, VGG19, Xception, and NASNetMobile. Each model was fine-tuned on the preprocessed dataset to extract high-level visual features. These features capture subtle stroke patterns, curvature, and spatial configurations, which are essential for distinguishing between visually similar Arabic letters—especially in children’s handwriting, where variability and shape ambiguity are more prominent.

Performance evaluations guided the selection of the most robust models to serve as base learners in the proposed ensemble framework. These models were chosen for their diverse architectural properties and complementary strengths. After training, each model generates a softmax probability distribution over the Arabic character classes, serving as the foundational input for the stacking ensemble. This multi-model setup ensures that a rich variety of feature perspectives is retained, laying the groundwork for a more accurate and robust final prediction.

#### 3.2.3. Stacking-Based Ensemble Learning

To further enhance the robustness and accuracy of the classification system, a stacking-based ensemble learning strategy was employed. Stacking is a hierarchical ensemble approach that combines the predictive strengths of multiple base models through a second-level learner, known as the meta-learner, which learns to optimally integrate their outputs [21]. This design enables the framework to leverage the complementary capabilities of diverse CNN architectures, thereby mitigating the weaknesses of individual models and improving overall generalization.

The proposed stacking ensemble integrates three high-performing convolutional neural networks—ConvNeXtBase, DenseNet201, and VGG16—through a fully connected meta-learner. Each base model contributes unique feature representations, ensuring diversity in learned patterns and reducing the risk of overfitting to specific handwriting styles. This multi-model strategy addresses performance variability across character classes, a well-documented challenge in ensemble systems where class-dependent behavior can significantly impact generalization performance [22]. By aggregating heterogeneous features, the ensemble produces a more balanced and reliable decision boundary, particularly suited for the diverse and inconsistent writing patterns found in children’s Arabic handwriting.

The meta-learner, implemented as a fully connected neural network, was trained on this stacked feature space to learn optimal fusion weights among the base predictions. Its architecture consists of two hidden layers with 128 and 64 neurons, both activated using ReLU, followed by a softmax output layer corresponding to the number of character classes. The meta-learner was trained using the same ground truth labels as the base models but operates on their softmax outputs rather than raw image data.

During inference, each input image is passed through the three base models to generate prediction vectors, which are concatenated and processed by the trained meta-learner to produce the final classification. This architecture enables the system to adaptively weight the contributions of each base learner, providing a dynamic and context-aware fusion that enhances predictive stability and confidence.

#### 3.2.4. Confidence Thresholding and Dynamic Optimization

Incorporating confidence estimation mechanisms has proven essential for building trustworthy and robust recognition systems [23]. To improve the reliability of the classification outcomes and reduce misclassifications due to uncertain predictions, a confidence-thresholding mechanism was integrated into the ensemble model’s decision-making process. Rather than accepting all predictions unconditionally, the system filters outputs based on a confidence score derived from the softmax probability of the predicted class. A prediction is accepted only if its associated confidence (i.e., the maximum softmax probability) exceeds a predefined threshold τ. This approach enables the system to reject low-confidence outputs, thereby enhancing robustness, particularly in scenarios involving ambiguous or visually similar Arabic characters.

Let *C* denote the total number of possible classes. For a given input sample $x_{i}$, the model produces a probability distribution over all classes, represented as $p_{i}=\left[p_{i1},p_{i2},\ldots ,p_{iC}\right]$. A prediction is accepted only when the highest class probability exceeds a predefined confidence threshold τ:

(1) $$\max_{j}\;p_{ij}\geq \tau .$$

This condition ensures that only predictions with sufficient confidence are retained, thereby improving the overall reliability of the classification process.

To avoid the pitfalls of arbitrary threshold selection, a dynamic optimization strategy was employed to determine the optimal threshold $\tau^{*}$. Furthermore, to ensure robustness against distribution shifts, $\tau^{*}$ was optimized on the combined validation sets from both datasets. This was achieved by evaluating a range of threshold values τ∈[0.5,0.99] on the validation set and computing performance metrics, such as the macro-averaged $F_{1}$ score, precision, and recall, for each. The threshold that yielded the highest $F_{1}$ score was selected as the operating point:

(2) $$\tau^{*}=\arg\max_{\tau \in T}F_{1}\left(\tau \right),$$

where *T* is the set of candidate thresholds, and $F_{1}\left(\tau \right)$ is the $F_{1}$ score at threshold τ.

This adaptive mechanism ensures a balanced trade-off between precision and recall and provides a mechanism for confidence calibration, enabling more reliable deployments of the model in real-world applications. The complete procedure for selecting the optimal threshold $\tau^{*}$ through dynamic optimization is summarized in Algorithm 1.
**Algorithm 1** Confidence Thresholding with Dynamic Optimization**Input:** Trained classifier f(·), combined validation set 𝒱, and candidate thresholds T={0.50,0.52,…,0.99}.**For each threshold**τ∈T:
(a)   Compute predictions on 𝒱 and keep only those with $\max_{j}p_{ij}\geq \tau$.(b)   Calculate rejection rate $\mathrm{rej}\_\mathrm{rate}=1-\frac{\mathrm{accepted}\;\mathrm{samples}}{N}$.(c)   If rej_rate>0.5, discard this threshold.(d)   Otherwise, compute macro-averaged Precision, Recall, and $F_{1}\left(\tau \right)$.**Select** the optimal threshold:$$\tau^{*}=\arg\max_{\tau \in T}F_{1}\left(\tau \right).$$

## 4. Experimental Results

### 4.1. Experimental Setup and Evaluation Metrics

All experiments were conducted using Google Colab Pro (Google LLC, Mountain View, CA, USA), which provided a high-performance cloud environment equipped with an NVIDIA A100 GPU, enabling efficient model training and evaluation. TensorFlow (Keras API) and scikit-learn were used to implement the deep learning models, stacking architecture, and evaluation procedures.

The proposed models were evaluated using the following standard metrics:

- Accuracy: The overall proportion of correct predictions.
- Precision: The proportion of correctly predicted positive instances among all predicted positives.
- Recall: The proportion of correctly predicted positives among all actual positives.
- F1 Score: The harmonic mean of precision and recall, particularly useful for imbalanced data.
- Expected Calibration Error (ECE): A measure of how well predicted confidence aligns with empirical accuracy. ECE is computed by grouping predictions into confidence bins and averaging the absolute differences between accuracy and confidence in each bin [24]. Temperature scaling [25] was applied as a post hoc calibration method to reduce miscalibration.
- McNemar’s test: A statistical test used to compare the error distributions of two classifiers evaluated on the same dataset, determining whether differences in their predictions are statistically significant.

Macro-averaging was employed for all metrics to treat each Arabic character class equally, ensuring a balanced evaluation despite potential class imbalance.

### 4.2. Hyperparameter Tuning

#### 4.2.1. Tuning of Base CNN Models

To ensure the optimal performance of each base learner, a systematic hyperparameter tuning process was conducted. This involved empirical validation using stratified training and validation splits from both the Hijja and Dhad datasets to promote reliable convergence, model stability, and strong generalization. All models were trained using the Nadam optimizer with a learning rate of $1\times 10^{-4}$, which was selected for its adaptive learning behavior and ability to reach stable convergence. The loss function was set to categorical cross-entropy, appropriate for the multi-class nature of Arabic character classification.

A batch size of 16 was used to balance computational efficiency with gradient estimation quality, and training was performed for a maximum of 50 epochs. To further enhance convergence and prevent overfitting, training included a set of callbacks: EarlyStopping with a patience of 10 epochs, ReduceLROnPlateau for dynamic learning rate adjustment, and ModelCheckpoint to save the best-performing weights. These hyperparameters were applied consistently across all base models, with minor adaptations when necessary, to ensure a fair and uniform evaluation within the ensemble framework.

#### 4.2.2. Hyperparameters for Ensemble Stacking

The stacking ensemble architecture was implemented using a fully connected neural network as the meta-learner, designed to integrate the probabilistic outputs of the selected base models. The meta-learner was trained using the Adam optimizer with a learning rate of $1\times 10^{-3}$, which was chosen based on preliminary tuning to ensure stable and efficient convergence. The categorical cross-entropy loss function was applied, consistent with the multi-class classification objective of Arabic character recognition. As input, the meta-learner received concatenated softmax probability vectors from the base learners, providing a rich and diverse representation for modeling inter-model dependencies.

Training was performed with a batch size of 32 for up to 10 epochs, which was sufficient to achieve convergence without overfitting. To further enhance prediction reliability, a dynamic confidence-thresholding mechanism was integrated. This process involved evaluating thresholds between 0.5 and 0.99 and selecting the optimal value ($\tau^{*}$) that maximized the macro-averaged $F_{1}$ score on the validation set. By applying this adaptive filtering strategy, the ensemble was able to reject uncertain predictions and produce more robust and trustworthy classification outcomes.

### 4.3. Transfer Learning Evaluation and Model Selection

**Figure 3:**
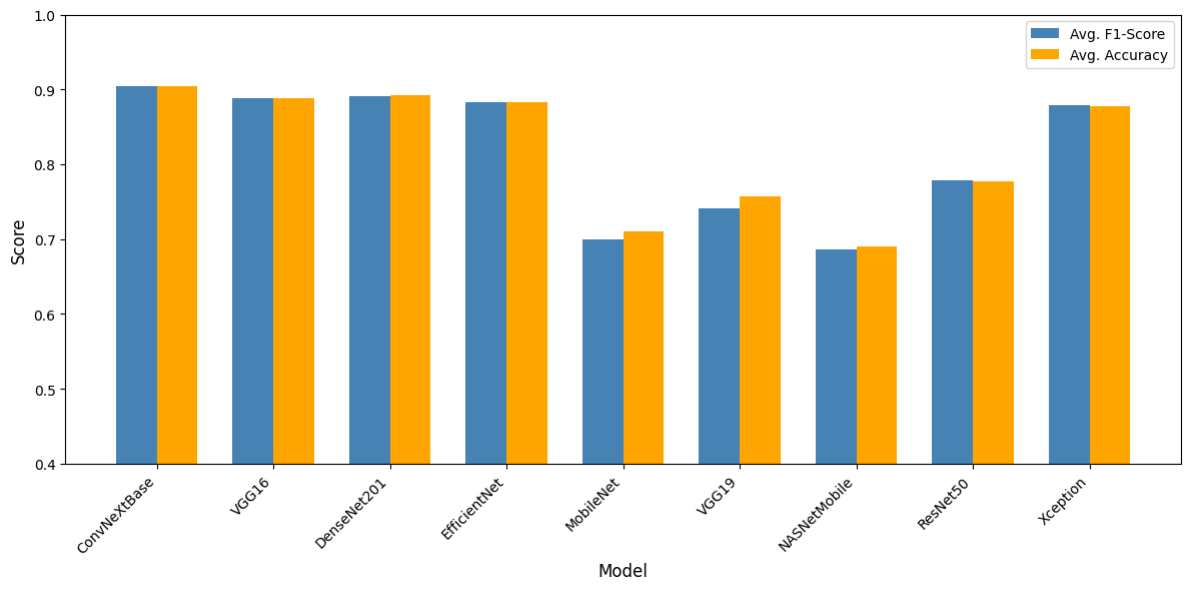
Average F_1_ score and accuracy comparison across evaluated models for both Hijja and Dhad datasets.

To identify the most effective base learners for the proposed stacking-based ensemble, a comprehensive evaluation was conducted across nine pre-trained convolutional neural network (CNN) architectures using transfer learning on both the Hijja and Dhad datasets. Each model was assessed based on a macro-averaged $F_{1}$ score and overall classification accuracy to ensure robust generalization across different handwriting sources. The results, summarized in Table 2 and illustrated in Figure 3, highlight distinct performance variations across architectures.

Among the evaluated models, ConvNeXtBase consistently achieved superior results, with the highest average accuracy of 90.42% and $F_{1}$ score of 90.38% across the two datasets. Its performance was both stable and robust, confirming its suitability as a primary component of the ensemble. DenseNet201 also produced competitive results, particularly excelling on the Dhad dataset (accuracy: 90.34%; $F_{1}$ score: 90.29%), contributing to an overall average accuracy of 89.18% and $F_{1}$ score of 89.12%.

Although VGG16 did not outperform the most advanced models in absolute terms, it delivered consistently reliable results across both datasets, with an average accuracy of 88.27% and $F_{1}$ score of 88.25%. Its architectural simplicity and regularization capabilities provide valuable diversity to the ensemble and help reduce the risk of overfitting.

To verify that the selected models contribute complementary behaviors, inter-model diversity was evaluated using pairwise error correlation and confusion overlap (Table 3). On the Hijja dataset, error correlations ranged from 0.547 to 0.562, with confusion overlap values between 0.425 and 0.441. Comparable patterns were observed on the Dhad dataset, where error correlations ranged from 0.546 to 0.583 and confusion overlap values ranged from 0.418 to 0.452. These moderate levels of agreement indicate that the base models do not consistently misclassify the same samples or confuse the same character classes. Consequently, ConvNeXtBase, DenseNet201, and VGG16 provide heterogeneous and complementary feature representations, supporting their integration within a stacking ensemble framework.

To ensure that the augmentation pipeline used in the transfer-learning evaluation did not introduce distortions to the Arabic characters, we conducted an ablation study, examining the effect of removing flips or applying them individually. Table 4 compares four settings: no flips, horizontal flips only, vertical flips only, and the proposed augmentation pipeline. Removing flips resulted in a consistent 1.5–3% reduction in accuracy and $F_{1}$ score, while horizontal and vertical flips yielded performance nearly identical to the proposed configuration. These results confirm that the flip-based augmentations used in this study do not introduce harmful artifacts and do not affect the comparative ranking of the evaluated CNN models.

Based on these findings, ConvNeXtBase, DenseNet201, and VGG16 were selected as the ensemble’s base models. This combination offers a balanced mix of modern representational strength, empirical robustness, and architectural diversity, enhancing the ensemble’s ability to generalize across diverse handwriting styles and improving recognition performance in challenging scenarios.

### 4.4. Stacking Ensemble Results and Confidence Threshold Optimization

This subsection presents a comprehensive evaluation of the proposed stacking ensemble, beginning with an ablation of fusion strategies and followed by analyses of computational efficiency, confidence thresholding, calibration, stability, and statistical significance.

#### 4.4.1. Fusion Strategy Ablation

Before applying confidence thresholding, we first evaluated the effectiveness of several ensemble fusion strategies to establish the suitability of the stacking meta-classifier. The tested methods included majority voting, soft averaging, a top-2 ensemble, and a top-3 ensemble. As shown in Table 5, all alternative fusion schemes yielded lower accuracy and $F_{1}$ scores than the proposed stacking approach on both the Hijja and Dhad datasets. Majority voting and soft averaging produced moderate performance but were unable to fully leverage the complementary representations learned by the individual CNNs. Likewise, the top-2 and top-3 ensembles underperformed, indicating that excluding any backbone reduces predictive robustness. These results, which are consistent with the inter-model diversity analysis, confirm that a learned meta-classifier provides a more effective integration mechanism for heterogeneous CNN features.

#### 4.4.2. Computational Complexity

To assess the computational feasibility of the proposed ensemble, Table 6 summarizes the number of trainable parameters, model size, FLOPs, and inference time for each component. Despite integrating three large-scale CNNs, the framework remains practical because the meta-classifier is lightweight (0.021M parameters) and adds negligible overhead. Overall, inference times remain within feasible limits, demonstrating that the stacking ensemble is computationally efficient for real-world handwriting recognition scenarios.

#### 4.4.3. Confidence Thresholding

To enhance prediction reliability, a confidence-thresholding mechanism was applied to the meta-learner’s outputs. A prediction was accepted only when its maximum softmax probability exceeded a threshold, τ. Candidate thresholds ranging from 0.5 to 0.99 were evaluated in 20 linearly spaced steps, excluding thresholds that rejected more than 50% of the validation samples. The optimal threshold was determined as $\tau^{*}=0.94$, selected according to the macro-averaged $F_{1}$ score on the combined validation sets.

As shown in Table 7, applying the optimized threshold (τ=0.94) improved accuracy, precision, and $F_{1}$ score across both datasets, with stronger gains observed on Hijja. Precision improvements exceeded recall, confirming that confidence filtering primarily reduces false positives. The consistent optimal threshold across datasets suggests robustness to distributional differences. For all experiments, the optimal threshold $\tau^{*}$ was computed exclusively on the validation set and was fixed prior to test-time evaluation to avoid data leakage. During optimization, predictions with maximum softmax probabilities below τ were treated as incorrect, ensuring that the $F_{1}$ score accurately reflects the balance between reliability and coverage. At the globally chosen threshold of τ=0.94, optimized from a single validation run, the test set rejection rate was 9.57% for Hijja (coverage: 90.43%) and 7.74% for Dhad (coverage: 92.26%), reflecting a favorable balance between sample retention and predictive reliability.

**Figure 4:**
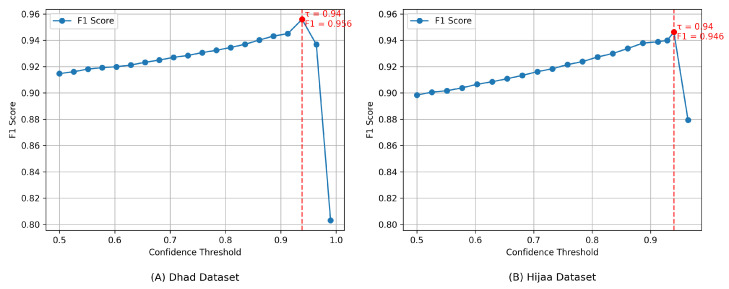
$F_{1}$ score vs. confidence threshold for the stacking ensemble on the (**A**) Dhad and (**B**) Hijja datasets, showing peak performance at τ=0.94.

Figure 4 illustrates the relationship between the confidence threshold and $F_{1}$ score. For both datasets, performance improves as the threshold increases, peaking at τ=0.94. Beyond this point, performance declines due to excessive sample rejection. This behavior validates the selected threshold and highlights its importance when high-confidence predictions are required.

#### 4.4.4. Calibration and Reliability Analysis

To further strengthen the uncertainty assessment, the expected calibration error (ECE) was computed before and after applying temperature scaling. As shown in Table 8, the optimal temperatures greater than one indicated initial overconfidence in the ensemble predictions. Temperature scaling substantially improved calibration quality for both datasets, reducing ECE from approximately 0.04–0.05 to below 0.01, corresponding to an 86% reduction for Hijja and a 91% reduction for Dhad, while preserving classification accuracy. Although calibration is not the primary focus of this study, these findings indicate that combining calibrated confidence estimates with threshold-based filtering enhances reliability for children’s handwriting recognition.

#### 4.4.5. Stability Across Random Seeds

To evaluate reproducibility, all experiments were repeated using five different random seeds. Table 9 reports the mean, standard deviation, and 95% confidence intervals for accuracy, precision, recall, $F_{1}$ score, and coverage, all computed on the test set and averaged across the five seeds. The small variances across seeds indicate that the ensemble is stable and not sensitive to initialization. Coverage values also remained consistent, demonstrating the robustness of the rejection mechanism under the optimized threshold.

#### 4.4.6. Statistical Significance Using McNemar’s Test

To determine whether the improvements over baseline CNNs were statistically meaningful, McNemar’s test was applied (Table 10). For both datasets, the number of instances where the ensemble corrected baseline errors (*c*) was substantially larger than the instances where the baseline outperformed the ensemble (*b*). All p-values were below 0.05, with most values being much smaller, indicating statistically significant or highly significant improvements. These findings confirm that the ensemble’s gains represent genuine increases in predictive robustness rather than random variation.

Together, these results reinforce the robustness, reliability, and statistically significant performance gains of the proposed stacking ensemble.

### 4.5. Per-Class Evaluation and Error Analysis

Table 11 presents the final performance of the proposed ensemble after applying the optimized confidence threshold (τ=0.94), providing a consolidated view of accuracy, precision, recall, and $F_{1}$ score and confirming the robustness of the approach on both Hijja and Dhad.

To further examine the model’s strengths and remaining challenges, we conducted a detailed per-class evaluation of the $F_{1}$ scores, as summarized in Table 12. This analysis offers character-level insight beyond the macro-averaged results and highlights patterns that inform future refinement.

Across both the Hijja and Dhad datasets, the model demonstrates outstanding recognition performance for several Arabic characters, particularly those with distinctive shapes and minimal structural ambiguity. Characters such as Alif أ ($F_{1}$ score: 99.11%, 97.36%), Sin س (98.20%, 97.31%), Shin ش (98.12%, 97.65%), Waw و (96.75%, 98.27%), and Ya ي (97.58%, 97.66%) achieved $F_{1}$ scores exceeding 97%. These results indicate that the combination of diverse CNN learners and stacking-based feature fusion effectively captures discriminative features even amid natural handwriting variability.

**Figure 5:**
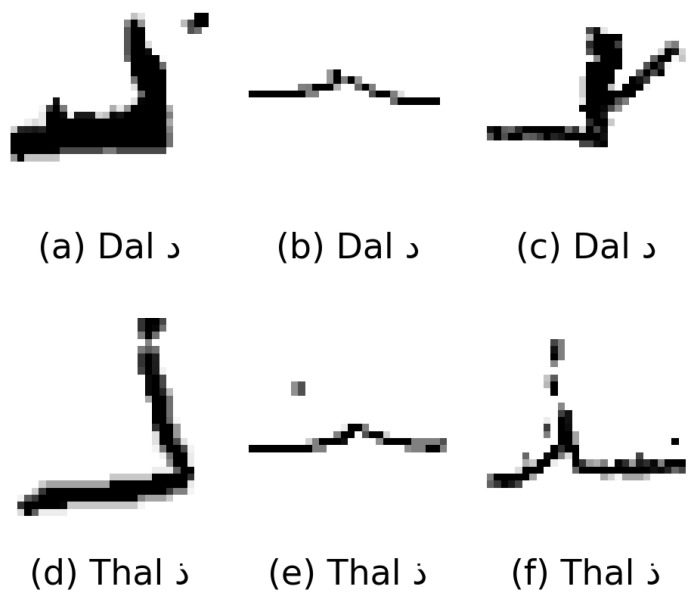
Examples of children’s handwritten samples for visually similar Arabic characters: (**a**–**c**) Dal د and (**d**–**f**) Thal ذ. The similarity, distinguished only by a single diacritical dot, often results in misclassifications in recognition systems.

However, two characters (Dal د and Thal ذ) remain among the most challenging across both datasets, with $F_{1}$ scores of 85.25% and 84.31% for Dal and 86.13% and 82.56% for Thal in the Hijja and Dhad datasets, respectively. The primary source of difficulty lies in their extreme visual similarity: the only distinguishing feature is a single diacritical dot, which is frequently omitted, misplaced, or distorted in children’s handwriting (Figure 5). Such subtle cues are easily masked by variations in stroke execution, making these classes difficult for both models and human annotators. When written in connected forms, these characters may also visually blend with adjacent strokes, further increasing the likelihood of misclassification.

Interestingly, several characters exhibit noticeable shifts in $F_{1}$ score performance between the Hijja and Dhad datasets. For instance, Ta ت drops by 4.3%, Tha ث by 3.1% and Ra ر by 4.7% in the Dhad dataset, which may reflect greater variability in handwriting style, increased noise, or sample imbalance in that collection. Conversely, characters such as Ha ح, Dad ض, and Ayn ع show slight performance gains in Dhad, suggesting that particular handwriting traits or clearer character formations may be more common in that dataset.

Taken together, the ensemble’s strong performance is driven by high accuracy on characters with distinctive and stable shapes, whereas its most systematic errors occur within visually similar character groups, particularly pairs such as Dal (د) and Thal (ذ), where small and often degraded diacritical marks are the sole distinguishing feature. Importantly, these failures are not arbitrary; they follow predictable patterns rooted in the structure of Arabic script, in which linguistic meaning is encoded in minute diacritics that are frequently unstable in children’s handwriting. Variations in performance across the Hijja and Dhad datasets can also be attributed to differences in handwriting variability, image quality, and class imbalance. Thus, although the ensemble represents a substantial improvement over individual CNNs, its remaining limitations reflect fundamental challenges inherent in handwritten Arabic recognition. These insights point directly to the need for future research focused on diacritic-sensitive representations and improved robustness to real-world noise.

Although the ensemble enhances robustness across most character classes, it does not incorporate a dedicated mechanism for modeling diacritical marks or fine-grained structural variations. A specialized dot-attention module or a multi-task branch for diacritic prediction represents a promising direction for future enhancement and may further reduce ambiguity among visually similar characters.

## 5. Comparison with Existing Studies and Discussion

This section positions the proposed stacking ensemble within the context of recent research on Arabic handwritten character recognition and discusses its performance, reliability, and methodological contributions relative to state-of-the-art approaches.

Table 13 presents a comparative analysis of the proposed stacking-based ensemble model against several state-of-the-art methods previously applied to the Hijja and Dhad datasets. Performance metrics include accuracy, precision, recall, and $F_{1}$ score, reported as percentages for consistency.

For the Hijja dataset, the proposed method significantly outperforms existing approaches. Notably, while Alheraki et al. [18] and Alwagdani et al. [17] report accuracy scores of 91% and 91.95%, respectively, our ensemble model achieves 95.13% accuracy and a macro-averaged $F_{1}$ score of 94.62%. These improvements reflect balanced gains in precision and recall, indicating strong generalization across all 29 character classes. The gap is particularly meaningful given that prior studies largely rely on single CNN architectures and do not incorporate uncertainty-aware mechanisms. In contrast, our stacking meta-learner and confidence thresholding jointly contribute to a more robust and reliable decision pipeline.

For the Dhad dataset, the proposed ensemble again surpasses prior results. AlMuhaideb et al. [20] reported an $F_{1}$ score of 94%, whereas our approach attains 95.59% with an accuracy of 96.14%. These findings highlight the advantage of combining diverse CNN backbones with softmax threshold optimization to mitigate misclassifications in noisier or more variable handwriting samples.

Recent studies have emphasized the importance of uncertainty calibration in deep learning models, particularly in settings where image quality or handwriting clarity varies. Neural networks can produce overconfident probability estimates, as demonstrated by Guo et al. [25]. Post hoc calibration techniques such as temperature scaling help align predicted confidence with the true likelihood of correctness. Within this context, the dynamic confidence thresholding employed in our framework serves as an effective uncertainty-aware mechanism by suppressing low-confidence predictions and improving decision reliability. This connection underscores the relevance of confidence-based filtering in children’s handwriting recognition, where ambiguity and handwriting irregularities are common.

Although the proposed ensemble demonstrates strong performance on each dataset individually, the model was not evaluated in a cross-dataset training–testing setting (e.g., training on Hijja and testing on Dhad). The two datasets exhibit notable distributional differences in writing environment, imaging conditions, stroke characteristics, and noise patterns. For this reason, the current study focuses on intra-dataset robustness. Extending the framework to cross-dataset or domain-adaptation scenarios represents an important direction for future research.

It is important to emphasize that the novelty of the proposed approach does not reside in the individual CNN backbones, which are well established in the literature, but rather in the way these models are strategically integrated within a confidence-weighted stacking ensemble. By leveraging the complementary representations learned by diverse pre-trained networks, the ensemble captures a broader range of discriminative cues that are particularly valuable for children’s handwriting, where stroke formation and diacritic placement are often inconsistent. The dynamic confidence-thresholding mechanism further introduces a practical uncertainty-aware component by filtering low-confidence predictions. Although the aim of this study is not to develop a new calibration algorithm, the integration of reliability-driven filtering with ensemble diversity has not been explored in prior studies on children’s Arabic handwriting and constitutes the core methodological contribution of this framework.

## 6. Conclusions

This study presents a confidence-based stacking ensemble that combines ConvNeXtBase, DenseNet201, and VGG16 via a meta-learner for recognizing children’s handwritten Arabic characters. A dynamic confidence threshold improves reliability by filtering uncertain predictions. The model achieves state-of-the-art results, with $F_{1}$ scores of 95.13% on the Hijja dataset and 95.59% on Dhad. Visually similar character pairs, such as Dal (د) and Thal (ذ), remain challenging due to missing or faint diacritics in children’s handwriting.

Several limitations remain. The model does not explicitly model Arabic diacritical marks, which are crucial yet often degraded. The Hijja and Dhad datasets also share similar demographic origins, potentially limiting style diversity. Furthermore, the low input resolution (32×32 pixels) constrains fine diacritic recognition, and the multi-CNN ensemble introduces higher computational costs. Future research will explore diacritic-aware attention, lightweight distillation, transformer architectures, and domain adaptation to enhance robustness and efficiency for real-world educational applications.

## Figures and Tables

**Table 1:** Examples of Arabic characters in different positions: beginning, middle, end, and isolated.

Letter	Beginning	Middle	End	Isolated
*Ayn* (ع)	عـ	ـعـ	ـع	ع
*Ba* (ب)	بـ	ـبـ	ـب	ب
*Mim* (م)	مـ	ـمـ	ـم	م
*Kaf* (ك)	ڪ	ـكـ	كـ	ك

**Table 2:** Performance comparison of pre-trained CNN models on Hijja and Dhad datasets. All metrics are reported in percentages (%).

Model	Hijja		Dhad		Average	
	Accuracy	F_1_ Score	Accuracy	F_1_ Score	Accuracy	F_1_ Score
ConvNeXtBase	90.22	90.19	90.61	90.57	90.42	90.38
DenseNet201	88.01	87.95	90.34	90.29	89.18	89.12
VGG16	88.27	88.23	89.50	89.44	88.89	88.84
EfficientNet	88.04	88.02	88.50	88.47	88.27	88.25
Xception	85.42	85.50	90.14	90.18	87.78	87.84
ResNet50	66.78	66.84	88.77	88.77	77.78	77.81
VGG19	61.59	58.49	89.78	89.76	75.69	74.13
MobileNet	64.67	63.64	77.33	76.30	71.00	69.97
NASNetMobile	49.48	48.69	88.62	88.58	69.05	68.63

**Table 3:** Inter-model diversity analysis based on pairwise error correlations and confusion overlap for the Hijja and Dhad datasets. Lower values indicate higher model complementarity.

Model Pair	Hijja Err. Corr.	Hijja Conf. Overlap	Dhad Err. Corr.	Dhad Conf. Overlap
ConvNeXt vs. DenseNet	0.5620	0.4386	0.5830	0.4518
ConvNeXt vs. VGG16	0.5473	0.4253	0.5461	0.4180
DenseNet vs. VGG16	0.5579	0.4406	0.5516	0.4244

**Table 4:** Effect of flip augmentations on model performance for Hijja and Dhad datasets. Values represent accuracy and F_1_ score (%).

Augmentation	Hijja			Dhad		
	ConvNeXt	DenseNet	VGG16	ConvNeXt	DenseNet	VGG16
No flip (Acc)	88.771	85.110	86.271	88.510	88.441	87.442
No flip (F_1_ score)	88.590	84.951	85.332	88.232	87.641	86.692
Horizontal only (Acc)	90.221	88.010	88.271	90.609	90.341	89.499
Horizontal only (F_1_ score)	90.190	87.951	88.232	90.572	90.289	89.442
Vertical only (Acc)	90.220	88.007	88.267	90.608	90.340	89.495
Vertical only (F_1_ score)	90.188	87.948	88.228	90.569	90.288	89.440
Proposed (Acc)	90.223	88.012	88.274	90.612	90.343	89.502
Proposed (F_1_ score)	90.193	87.954	88.232	90.574	90.293	89.443

**Table 5:** Ablation study comparing ensemble fusion methods on the Hijja and Dhad datasets. Values represent Accuracy, Precision, Recall, and F_1_-score (%).

Method	Hijja				Dhad			
	Accuracy	Precision	Recall	F_1_ Score	Accuracy	Precision	Recall	F_1_ Score
Majority Vote	92.89	91.71	91.65	91.64	93.96	92.54	92.32	92.40
Soft Averaging	92.89	91.33	91.29	91.27	93.34	92.99	92.81	92.87
Top-2 Ensemble	91.90	90.36	90.45	89.36	92.58	91.23	91.92	92.03
Top-3 Ensemble	92.25	91.33	91.29	91.27	93.34	92.99	92.81	92.87

**Table 6:** Computational complexity of the backbone models and meta-classifier for a 32×32×3 input. Model size is reported in megabytes (MBs), FLOPs in gigaflops (GFLOPs), and inference time in milliseconds per sample.

Model	Params (M)	Size (MB)	FLOPs (GFLOPs)	Time (Hijja) (ms)	Time (Dhad) (ms)
ConvNeXtBase	87.60	334.17	0.621	88.88	67.82
DenseNet201	18.39	70.13	0.177	91.95	79.97
VGG16	14.73	56.20	0.627	82.09	67.27
Meta-classifier	0.021	0.08	0.00004	85.60	61.48

**Table 7:** Performance comparison of the meta-learner with and without confidence thresholding.

Dataset	Threshold (τ)	Accuracy (%)	Precision (%)	Recall (%)	F_1_ Score (%)
Hijja	0.94	95.13	95.07	94.28	94.62
	None	93.18	92.73	92.71	92.69
Dhad	0.94	96.14	95.88	95.38	95.59
	None	94.34	94.04	93.73	93.86

**Table 8:** Temperature scaling calibration performance for the proposed ensemble. ECE values are reported before and after calibration, along with the corresponding percentage reduction.

Dataset	Optimal *T*	ECE Before	ECE After	Reduction (%)
Hijja	1.4625	0.0435	0.0061	86.1% ↓
Dhad	1.6772	0.0470	0.0040	91.4% ↓

↓ indicates a reduction in ECE after temperature scaling calibration.

**Table 9:** Prediction performance across five random seeds. Values represent mean ± standard deviation and 95% confidence intervals (CIs).

Dataset	Accuracy (%)	Precision (%)	Recall (%)	F1 Score (%)	Coverage (%)
Hijja	95.30 ± 0.12	94.97 ± 0.12	94.77 ± 0.12	94.85 ± 0.11	88.99 ± 0.23
	(CI: 95.20–95.41)	(CI: 94.87–95.08)	(CI: 94.66–94.87)	(CI: 94.75–94.95)	(CI: 88.79–89.19)
Dhad	96.03 ± 0.14	95.42 ± 0.44	94.95 ± 1.11	95.09 ± 0.90	87.83 ± 0.68
	(CI: 95.91–96.16)	(CI: 95.04–95.80)	(CI: 93.98–95.91)	(CI: 94.31–95.88)	(CI: 87.24–88.42)

**Table 10:** McNemar’s statistical significance test comparing baseline CNNs with the proposed meta-classifier. *b* = Cases where the baseline was correct and the meta-classifier was wrong; *c* = cases where the baseline was wrong and the meta-classifier was correct. All reported *p*-values below 0.05 indicate statistically significant improvements.

Dataset	Baseline Model	*b*	*c*	*p*-Value	Significance
Hijja	ConvNeXtBase	4	21	$1.37\times 10^{-3}$	Significant
	DenseNet201	67	317	$5.43\times 10^{-37}$	Highly significant
	VGG16	55	263	$3.75\times 10^{-31}$	Highly significant
Dhad	ConvNeXtBase	4	35	$1.56\times 10^{-6}$	Significant
	DenseNet201	83	219	$7.95\times 10^{-15}$	Highly significant
	VGG16	101	306	$4.89\times 10^{-24}$	Highly significant

**Table 11:** Final performance summary of the proposed model on the Hijja and Dhad datasets using the optimized confidence threshold (τ=0.94).

Dataset	Accuracy (%)	Precision (%)	Recall (%)	$F_{1}$ Score (%)
Hijja	95.13	95.07	94.28	94.62
Dhad	96.14	95.88	95.38	95.59

**Table 12:** Per-class F_1_ score (%) comparison between Hijja and Dhad datasets.

Class	Arabic Letter	F_1_ Score Hijja (%)	F_1_ Score Dhad (%)
Alif	ا	99.11	97.36
Ba	ب	98.05	96.78
Ta	ت	98.01	93.71
Tha	ث	96.86	93.75
Jeem	ج	97.40	96.48
Haa	ح	94.01	95.66
Kha	خ	95.57	92.26
Dal	د	85.25	84.31
Thal	ذ	86.13	82.56
Ra	ر	98.07	93.38
Zay	ز	96.05	95.03
Sin	س	98.20	97.31
Shin	ش	98.12	97.65
Sad	ص	93.18	93.92
Dad	ض	93.85	95.05
Da	ط	96.46	96.26
Za	ظ	96.89	97.29
Ayn	ع	92.70	95.64
Gayn	غ	95.67	94.07
Fa	ف	93.66	93.42
Qaf	ق	96.60	94.67
Kaf	ك	96.28	96.57
Lam	ل	96.85	94.51
Mim	م	95.70	93.80
Noon	ن	95.04	91.07
Ha	هـ	95.15	97.57
Waw	و	96.75	98.27
Ya	ي	97.58	97.66
Hamza	ء	96.25	94.01
Macro-Average	–	94.62	95.59

**Table 13:** Performance comparison of existing methods with the proposed model on the Hijja and Dhad datasets.

Dataset	Method	Accuracy (%)	Precision (%)	Recall (%)	F_1_ Score (%)
Hijja	Altwaijry et al. [16]	88.00	88.00	88.00	88.00
	Alheraki et al. [18]	91.00	91.00	91.00	91.00
	Alshehri [26]	88.24	91.40	91.40	91.50
	AlMuhaideb et al. [20]	87.81	89.00	89.00	89.00
	Alwagdani et al. [17]	91.95	92.07	91.91	91.93
	**Proposed Method**	**95.13**	**95.07**	**94.28**	**94.62**
Dhad	AlMuhaideb et al. [20]	93.59	94.00	94.00	94.00
	**Proposed Method**	**96.14**	**95.88**	**95.38**	**95.59**

Bold values indicate the best performance among the compared methods.

## Data Availability

The datasets used in this study are publicly available. The Hijja dataset for children’s Arabic handwriting can be accessed at https://github.com/israksu/Hijja2 (accessed on 3 April 2025), and the Dhad dataset for children’s handwritten Arabic characters can be accessed at https://github.com/daadturki1/Dhad/tree/main/Dhad_Dataset (accessed on 3 April 2025). No new datasets were created during this study.

## Abbreviations

The following abbreviations are used in this manuscript:

AHR	Arabic Handwriting Recognition;
CNN	Convolutional Neural Network;
ReLU	Rectified Linear Unit;
SVM	Support Vector Machine;
τ	Confidence Threshold;
$\tau^{*}$	Optimized Confidence Threshold;
Hijja	Arabic Children’s Handwriting Dataset 1;
Dhad	Arabic Children’s Handwriting Dataset 2.
